# Association between coffee intake and frailty among older American adults: A population-based cross-sectional study

**DOI:** 10.3389/fnut.2023.1075817

**Published:** 2023-02-02

**Authors:** Shuo Pang, Guangrui Miao, Yuanhang Zhou, Mingxuan Duan, Linpeng Bai, Xiaoyan Zhao

**Affiliations:** Department of Cardiology, The First Affiliated Hospital of Zhengzhou University, Zhengzhou, China

**Keywords:** frailty, caffeinated coffee, decaffeinated coffee, dose-response, National Health and Nutrition Examination Survey

## Abstract

**Objective:**

We aimed to investigate the association between coffee consumption and frailty in older American adults. We focused on individuals at higher frailty risk, such as women, ethnic minorities, smokers, and those with obesity and insufficient physical activity.

**Methods:**

The data of 8,087 individuals aged over 60 years from the 2007–2018 National Health and Nutrition Examination Surveys were used for this cross-sectional study. The coffee drinks were classified into two categories: caffeinated and decaffeinated. Frailty was measured using the 53-item frailty index. Weighted binary logistic regression was used to evaluate the association between coffee intake and frailty risk. Restricted cubic spline models were used to assess the dose–response relationship between caffeinated coffee intake and frailty.

**Results:**

Among the 8,087 participants, 2,458 (30.4%) had frailty. Compared with those who reported no coffee consumption, the odds ratios [ORs; 95% confidence intervals (CIs)] of total coffee consumption > 498.9 (g/day) were 0.65 (0.52, 0.79) in the fully adjusted model. Compared with those who reported no caffeinated coffee consumption, the ORs (95% CIs) of total coffee consumption > 488.4 (g/day) were 0.68 (0.54, 0.85) in the fully adjusted model. Compared with those who reported no decaffeinated coffee consumption, the ORs (95% CIs) of total coffee consumption > 0 (g/day) were 0.87 (0.71, 1.06) in the fully adjusted model. Nonlinear associations were detected between total coffee and caffeinated coffee consumption and frailty. In the subgroup analyses by smoking status, the association between coffee consumption and the risk of frailty was more pronounced in non-smokers (P for interaction = 0.031).

**Conclusion:**

Caffeinated coffee consumption was independently and nonlinearly associated with frailty, especially in non-smokers. However, decaffeinated coffee consumption was not associated with frailty.

## 1. Introduction

Frailty refers to a state in which the decline of physiological reserves in older adults leads to increased vulnerability and decreased ability to resist stress (1). In older age, frailty is common (2) and leads to a higher risk of adverse health outcomes, including falls, hospitalization, and mortality (3). In the context of population aging, the burden of frailty and its costs to individuals and society are concerning (4). Therefore, it is essential to screen, prevent, and comprehensively treat frailty.

Fried’s phenotype and frailty index models are the two main instruments used to measure frailty (5). The phenotype model (3) defines frailty as meeting three or more of the five physical criteria. The frailty index model, developed by Rockwood et al. (6), is composed of a long checklist, including cognitive deficits, psychosocial factors, chronic diseases, and other geriatric signs and symptoms; the index is shown as the ratio of accumulated acquired deficits to all potential deficits. These two scoring models are complementary (5).

Although frailty is a complex and multifactorial process, nutritional factors play an important role in its occurrence and development (7). Moreover, nutrition is an easily modulated factor (8). Coffee is one of the most commonly consumed beverages worldwide and is believed to have many benefits. Studies have demonstrated that coffee consumption can reduce the risk of multiple diseases, including diabetes (9), cardiovascular disease (10), and liver disease (11). However, whether coffee consumption is associated with frailty remains unclear. To our knowledge, only three studies have examined the relationship between coffee intake and the risk of frailty, and their results were inconsistent. One prospective study indicated that coffee intake was not associated with frailty phenotype or disability in a Spanish population (12). Two cross-sectional studies found a significant association between coffee and frailty in Taiwan and Japan, respectively; however, coffee was only a small part of the research (13, 14). All the above-mentioned studies used the phenotype frailty model, and none examined the dose–response relationship between coffee and frailty. In our study, we used a 53-item frailty index and focused on individuals at higher frailty risk, such as women, ethnic minorities, smokers, and those with obesity and insufficient physical activity (15), because in such individuals, the effects of coffee may be more evident. Therefore, this study aimed to investigate the association between total, decaffeinated, and caffeinated coffee consumption and frailty index by analyzing data from the National Health and Nutrition Examination Survey (NHANES) 2007–2018.

## 2. Materials and methods

### 2.1. Data source and study population

**Figure 1:**
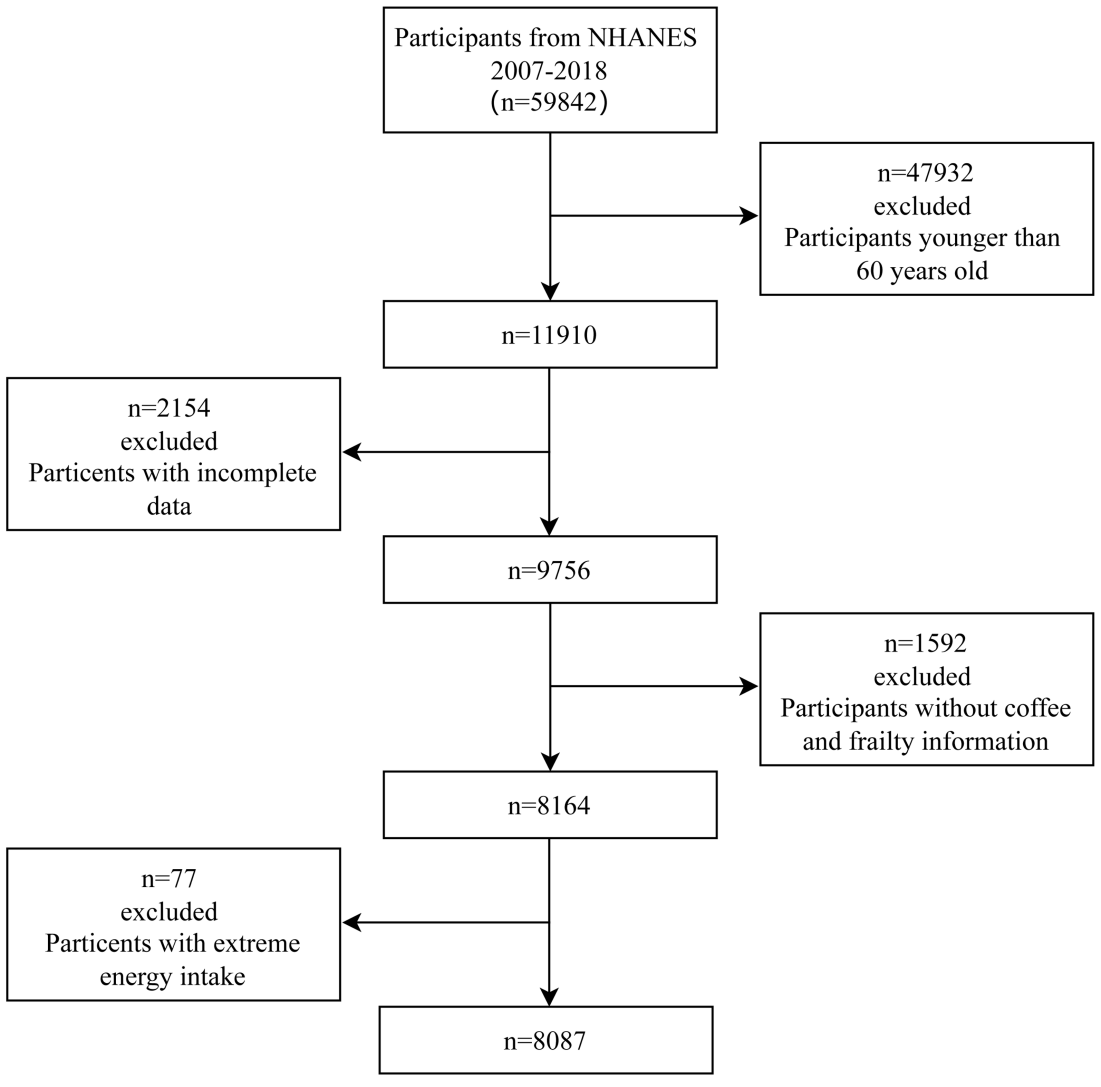
Flowchart for the selection of eligible participants.

We used data from 2007 to 2018 of the NHANES, a cross-sectional medical examination survey conducted by the Centers for Disease Control and Prevention of America. Using a complex stratified multi-stage sampling design, the NHANES evaluates the health status of a representative sample of the non-institutional United States population. NHANES 2007–2018 had data from 59,842 participants; among them, 11,910 were aged ≥ 60 years. A total of 8,164 participants were included in the analyses after excluding those who did not have complete coffee intake and frailty queries or had missing data for any other covariate. In addition, participants with extreme total energy intakes of <500 or >8,000 kcal/day for males and <500 or >5,000 kcal/day for females were excluded. Thus, 8,087 individuals were enrolled in this study (Figure 1).

### 2.2. Coffee consumption assessment

In all segments of the NHANES 2007–2018, coffee intake was reported in two 24-h dietary recall interviews. The first dietary recall interview was conducted in person, and the second interview was conducted *via* telephone call 3–10 days later. The United States Department of Agriculture Dietary Sources of Nutrients Database was used to identify coffee intake, and the first three numbers in the food code for coffee are “921”. In this study, one cup of coffee was equal to 6 ounces or 170 g. We used the average coffee intake from the two 24-h recalls for these analyses. We divided coffee drinks into two categories: caffeinated and decaffeinated. Aside from those who reported no coffee consumption, the total coffee consumption was divided into tertiles, resulting in four categories: (1) 0 g/day, (2) 1–270.5 g/day, (3) 270.5–498.9 g/day, and (4) >498.9 g/day. Excluding those who reported no caffeinated coffee consumption, caffeinated coffee consumption was divided into tertiles, resulting in four categories: (1) 0 g/day, (2) 1–253.3 g/day, (3) 253.3–488.4 g/day, and (4) >488.4 g/day. Decaffeinated coffee consumption was divided into two groups: (1) 0 g/day and (2) >0 g/day.

### 2.3. Frailty index assessment

Frailty was assessed in all cohorts using the standard procedure proposed by Searle et al. (16). Suitable traits for frailty index inclusion should be health deficits. These characteristics must be easily accessible, cover multiple systems, and increase their risk with age. The frailty index ranges from 0 to 1, and the degree of frailty increases with an increasing frailty value. Frailty was defined as a frailty index > 0.21 (17, 18). The frailty index included 53 variables (18), all of which were available in the NHANES database. The index system included cognition (one item), dependence (20 items), depressive symptoms (seven items), comorbidities (13 items), hospital utilization and access to care (five items), physical performance and anthropometry (one item), and laboratory values (six items). An explanation of the frailty index variables and their scores is provided in Supplementary Table 1.

### 2.4. Covariates

In addition to decaffeinated and caffeinated coffee intake, our study included several potential covariates: age, sex, ethnicity (Mexican American, non-Hispanic white, non-Hispanic black, Hispanic, and other races), education level (below high school, high school, and above high school), marital status (married/living with partner and never married /divorced/widowed/separated), and smoking status (never: smoked < 100 cigarettes in life; former: smoked > 100 cigarettes in life and currently not at all; now: smoked > 100 cigarettes in life and smoke some days or every day), body mass index (BMI), total energy intake, poverty-income ratio (the ratio of family income to the poverty threshold), hypertension, diabetes, coronary heart disease, stroke, and physical activity (inactive, low, moderate, and high). Physical activity was categorized according to the level of metabolic equivalent of task (MET): inactive (0 MET-min activity/week), low (1–499 MET-min activity/week), moderate (500–1,000 MET-min activity/week), and high (>1,000 MET-min activity/week) (19, 20).

### 2.5. Statistical analyses

We combined six 2-year segments (2007–2008, 2009–2010, 2011–2012, 2013–2014, 2015–2016, and 2017–2018) of the NHANES data into a single dataset and removed participants with missing data. The participants were divided into the frailty and non-frailty groups. Continuous variables were expressed as mean (standard error). The *t*-test was used to compare differences in continuous variables with a normal distribution. Categorical variables were expressed as percentages. The Chi-square test was used to compare the differences in categorical variables. Binary logistic regression analyses were used to estimate the odds ratios (ORs) and 95% confidence intervals (CIs) for the association between total, caffeinated, and decaffeinated coffee consumption and frailty risk. Model 1 was not adjusted for confounders. Model 2 was adjusted for age and sex. Model 3 was adjusted for ethnicity, educational level, marital status, poverty-to-income ratio, BMI, smoking status, total energy intake, hypertension, diabetes, coronary heart disease, stroke, and physical activity based on Model 2. To examine linear trends, the median value of coffee consumption within each coffee intake category was modeled as a continuous variable. Subgroup analyses were performed based on sex, ethnicity, smoking status, BMI, and physical activity. We assessed whether the frailty risk varied among the subgroups using likelihood-ratio tests, which compared the models with and without relevant interaction terms. We further used a restricted cubic spline curve with three knots at the 10th, 50th, and 90th percentiles of caffeinated coffee to assess the dose–response relationship between caffeinated coffee intake and frailty in the logistic regression Model 3. Sampling weights were used in all analyses, accounting for the complex, multi-stage sampling design of the NHANES. The sampling weights were calculated by dividing the 2-year sample weights by the number of 2-year segments in these analyses. All statistical analyses were performed using the statistical programming language, R software (version 4.2.0). A two-sided *p*-value ≤ 0.05 was considered statistically significant.

## 3. Results

The population characteristics according to frailty status are presented in Table 1. Among the study participants, 30.4% were frail. The mean ages of the participants without and with frailty were 68.72 (standard error = 0.13) and 71.43 (standard error = 0.22) years, respectively. Frail participants had a higher percentage of females, lower educational level, widowed/divorced/separated/never married marital status, lower poverty-income ratio, higher BMI, hypertension, diabetes, coronary heart disease, and stroke. Frail individuals had a lower consumption of coffee and caffeinated coffee, lower total energy intake, and a lower percentage of non-Hispanic white, non-smokers, and moderate and high physical activity.

**Table 1:** Characteristics of the study subjects according to frailty (weighted *N* = 46,266,020).

	Total	Non-frail	Frail	*P-*value
Unweighted *N*	8,087	5,629	2,458	
Age (years)	69.41 (0.11)	68.72 (0.13)	71.43 (0.22)	<0.001
Sex (%)				<0.001
Female	54.65	53.21	58.78	
Male	45.35	46.79	41.22	
Ethnicity (%)				<0.001
Mexican American	3.53	3.11	4.75	
Non-Hispanic White	80.74	82.37	76.03	
Non-Hispanic Black	7.95	7.24	10.01	
Other Hispanic	3.23	3.04	3.76	
Other race	4.56	4.25	5.46	
Educational level (%)				<0.001
Below high school	6.3	4.59	11.25	
High school	34.63	31.49	43.7	
Above high school	59.07	63.93	45.06	
Material status (%)				<0.001
Widowed/divorced/separated/never married	35.04	31.19	46.17	
Married/living with partner	64.96	68.81	53.83	
Smoke (%)				<0.001
Never	49.07	51.46	42.17	
Former	40.59	39.08	44.93	
Current	10.34	9.46	12.9	
Poverty-income ratio (%)				<0.001
<1	8.9	6.46	15.94	
≥1	91.1	93.54	84.06	
BMI (%)				<0.001
<25 kg/m^2^	24.35	26.9	16.99	
25–30 kg/m^2^	35.7	37.33	30.98	
>30 kg/m^2^	39.95	35.77	52.03	
Hypertension (%)	67.69	61.71	84.95	<0.001
Coronary heart disease (%)	10.32	5.87	23.17	<0.001
Diabetes (%)	39.1	32.63	57.8	<0.001
Stroke (%)	7.41	3.44	18.87	<0.001
Physical activity (%)				<0.001
Inactive	32.16	25.07	52.65	
Insufficient	14.23	14.34	13.93	
Moderate	11.46	12.7	7.87	
High	42.14	47.89	25.55	
Coffee intake (g/day)	403.32 (9.01)	418.59 (10.77)	359.24 (10.98)	<0.001
Caffeinated coffee intake (g/day)	332.38 (8.06)	347.22 (9.88)	289.55 (9.45)	<0.001
Decaffeinated coffee intake (g/day)	70.94 (4.06)	71.37 (4.91)	69.69 (7.43)	0.85
Total energy intake (kcal/day)	1916.99 (14.84)	1944.66 (19.23)	1837.11 (22.52)	<0.001

Table 2 shows the associations between frailty and total, caffeinated, and decaffeinated coffee intake. Compared with those who reported no coffee consumption, the ORs (95% CIs) of total coffee consumption > 498.9 (g/day) were 0.66 (0.55, 0.78) in the crude analysis, 0.68 (0.56, 0.81) in Model 2, and 0.65 (0.52, 0.79) in Model 3. Compared with those who reported no caffeinated coffee consumption, the ORs (95% CIs) of total coffee consumption >488.4 (g/day) were 0.66 (0.56, 0.79) in the crude analysis, 0.72 (0.60, 0.85) in Model 2, and 0.68 (0.54, 0.85) in Model 3. Compared with those who reported no decaffeinated coffee consumption, the ORs (95% CIs) of total coffee consumption >0 (g/day) were 1.00 (0.83, 1.21) in the crude analysis, 0.89 (0.74, 1.07) in Model 2, and 0.87 (0.71, 1.06) in Model 3.

**Table 2:** Weighted odds ratios (95% confidence intervals) for the association between coffee, caffeinated coffee, and decaffeinated coffee consumption and frailty.

Variable	Frailty			
	Case/participants	Model 1	Model 2	Model 3
Total coffee consumption (g/day)				
0	716/2,195	ref	ref	ref
0–270.5	632/1,967	0.99 (0.83, 1.19)	0.91 (0.75, 1.10)	0.87 (0.70, 1.07)
270.5–498.9	582/1,961	0.88 (0.74, 1.03)	0.81 (0.68,0.96)^*^	0.80 (0.64, 0.99)^*^
>498.9	528/1,964	0.66 (0.55, 0.78)^**^	0.68 (0.56, 0.81)^**^	0.65 (0.52, 0.79)^**^
P for trend		<0.01	<0.01	<0.01
Caffeinated coffee consumption (g/day)				
0	1,017/3,104	ref	ref	ref
0–253.3	526/1,665	1.04 (0.89, 1.23)	1.00 (0.84, 1.18)	0.96 (0.80, 1.16)
253.3–488.4	474/1,669	0.83 (0.72, 0.96)^*^	0.81 (0.70, 0.94)^*^	0.84 (0.69, 1.02)
>488.4	441/1,649	0.66 (0.56, 0.79)^**^	0.72 (0.60, 0.85)^**^	0.68 (0.54, 0.85)^**^
P for trend		<0.01	<0.01	<0.01
Decaffeinated coffee consumption (g/day)				
0	1,978/6,593	ref	ref	ref
>0	480/1,494	1.00 (0.83, 1.21)	0.89 (0.74, 1.07)	0.87 (0.71, 1.06)
P for trend		0.98	0.22	0.16

Model 1: crude model; Model 2: adjust for age and sex; Model 3: adjust for age, sex, ethnicity, educational level, marital status, poverty-to-income ratio, BMI, smoking status, total energy intake, hypertension, diabetes, coronary heart disease, stroke, physical activity.

* *p* < 0.05.

** *p* < 0.01.

**Figure 2:**
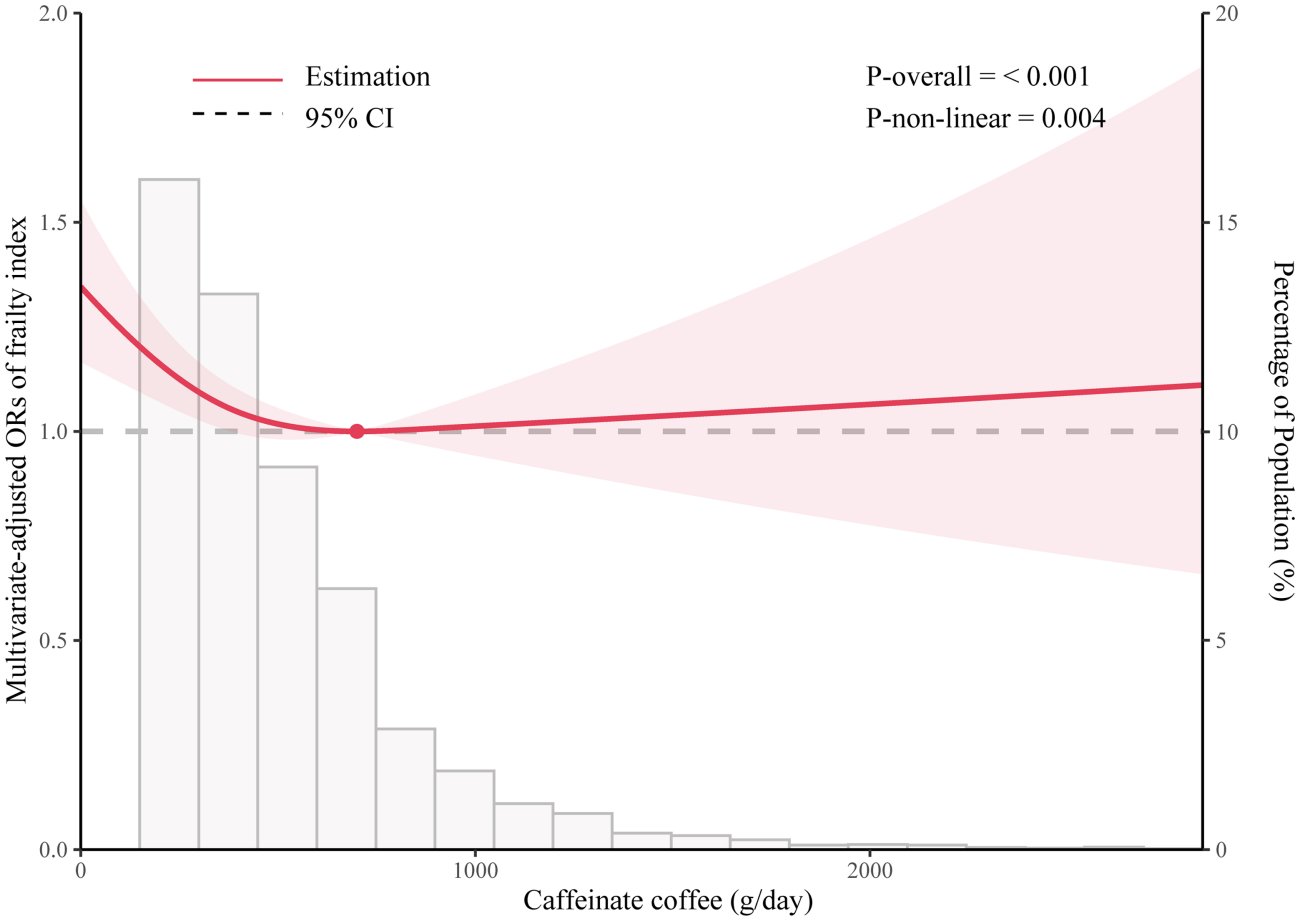
Dose–response relationship between caffeinated coffee intake and frailty.

**Figure 3:**
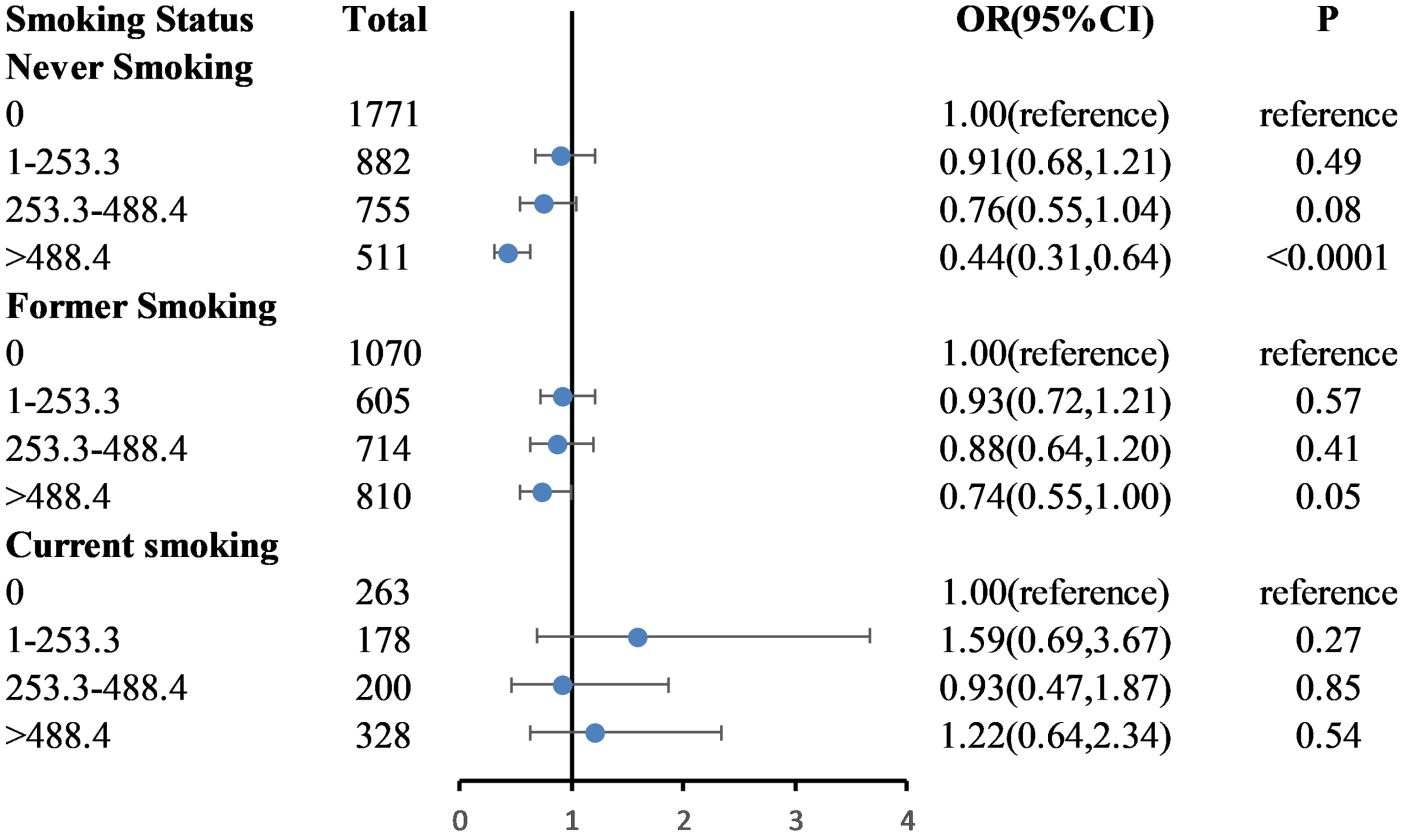
Weighted odds ratios (95% confidence intervals) for the association between caffeinated coffee and frailty by smoking status subgroups. The multivariable model is age, sex, ethnicity, educational level, marital status, poverty-to-income ratio, BMI, total energy intake, hypertension, diabetes, coronary heart disease, stroke, and physical activity.

Figure 2 shows the dose–response relationship between caffeinated coffee intake and frailty based on Model 3. A nonlinear relationship was found between caffeinated coffee consumption and frailty risk (P-nonlinear = 0.004). The plot showed a substantial reduction in the risk of frailty within the lower range of caffeinated coffee consumption, which reached the lowest risk at approximately 699.92 g/day and then increased thereafter. A statistically significant interaction was noted between caffeinated coffee and smoking status for the frailty index (P for interaction = 0.031; Figure 3). Associations between caffeinated coffee intake and the risk of frailty did not significantly differ by sex, ethnicity, physical activity, and BMI (all P for interaction > 0.05; Supplementary Table 2).

## 4. Discussion

This large cross-sectional study demonstrated that in a nationally representative sample of Americans aged > 60 years, total and caffeinated coffee consumption were significantly associated with the frailty index, and nonlinear dose–response relationships were also detected. No significant association was observed between decaffeinated coffee consumption and the frailty index. Subgroup analyses showed that a high level of caffeinated coffee consumption was associated with frailty in never/former smokers but not in current smokers.

Previous studies of the association between coffee consumption and the risk of frailty have yielded inconsistent results. Machado et al. (12) found that coffee intake was not associated with frailty or disability in a prospective analysis of data on older populations. Unlike our study, this survey was conducted in Spain. In Spain, unfiltered coffee is the most consumed type of coffee, in contrast to the United States, where coffee is mainly filtered. The diterpenes cafestol and kahweol in unfiltered coffee have been found to increase plasma levels of total cholesterol and triglycerides, which may be harmful to the cardiovascular system, which is closely related to frailty. Kobayashi et al. (13) found that dietary habits with high total antioxidant capacity, such as coffee consumption, showed a stronger inverse association with frailty among older Japanese women. Huang et al. (14) showed that participants with frailty had significantly lower daily coffee consumption. However, in these two studies that showed a significant association between coffee and frailty, coffee was only a small part of the research, and the effects of doses and different types of coffee on the risk of frailty were not explored in depth. In addition, all these studies used the frailty phenotype to define frailty. Our study is the first to assess the association between coffee consumption and frailty using the frailty index, which contains 53 deficits covering multi-systems. Compared with assessing physical signs and symptoms in the five-item phenotype models, the frailty index in this study allowed assessing the association between coffee intake and frailty based on an in-depth geriatric assessment. However, considering the limitations of cross-sectional studies, the role of caffeinated coffee on the frailty index should be further confirmed in longitudinal studies.

The mechanisms underlying coffee consumption and the risk of frailty are not fully understood; however, there are several possibilities. First, older people often have a poor antioxidant status, and frailty may result from tissue damage caused by oxidative stress and inflammation (21, 22). Coffee has been shown to contribute to a large proportion of the daily intake of dietary antioxidants, greater than tea, fruit, and vegetables (23). It contains caffeine, phenolics, and other bioactive compounds that are beneficial for health (24); these ingredients are potent antioxidants that protect the body from the harmful effects of free radicals (25). Second, coffee consumption is related to a lower risk of chronic diseases, including cardiovascular disease, metabolic disease, stroke, and certain cancers (26, 27), which, in turn, are the main components of the frailty index (6). Finally, coffee has been shown to induce autophagy, improve insulin sensitivity, increase glucose uptake, slow the progression of sarcopenia, and maintain muscle mass (28, 29), which are closely related to physical function and frailty. Thus, coffee may indirectly reduce the risk of frailty by slowing age-related sarcopenia and improving muscle function (12).

There is an increasing interest in the beneficial effects of decaffeinated coffee and its non-caffeinated compounds (30). Non-caffeine compounds, such as phenolic acids, not only have anti-inflammatory and antioxidant effects (31, 32) but also protect and prevent systemic diseases, such as cardiovascular disease, diabetes, and cancer (30). In our study, we divided coffee into caffeinated and decaffeinated types to determine the components associated with frailty. However, no significant association between decaffeinated coffee consumption and frailty was found in our study, which may indicate that caffeine plays a major role in the prevention of frailty.

Previous studies have reported sex differences in the effects of caffeine on various diseases, including fracture, dementia, and Parkinson’s disease (26, 33, 34). Relling et al. (35) found that xanthine oxidase activity was higher in healthy women than in men after consuming the same low dose of caffeine. Temple et al. (36) showed that sex differences in response to caffeine may be mediated by changes in circulating steroid hormone levels. In addition, women are more prone to frailty than men (37). In the current study, the prevalence of frailty was 58.6% among females and 41.2% among males. However, our results did not support a potential sex disparity for the effect of coffee consumption on the risk of frailty. Further studies are required to confirm these results.

Smokers tend to have an unhealthy lifestyle (38), and the proportion of smokers among frail individuals is higher. Studies have demonstrated a strong positive association between smoking and coffee consumption (39). Due to the known harmful effects of smoking and the widespread interest in the health effects of coffee (26), it is important to assess the association between coffee consumption and the risk of frailty according to smoking status. After adjusting for confounding factors, our study found that a higher caffeinated coffee intake was not associated with a reduced risk of frailty among current smokers. It is possible that the detrimental effects of smoking outweigh the positive effects of caffeinated coffee intake. Consequently, this association between coffee consumption and frailty may only be detectable in persons who do not smoke at present.

Our study has several advantages. A major strength of our study is the large, nationally representative United States population sample from the NHANES database. In addition, the dose–response and subgroup analyses were used to assess the associations. Moreover, to our knowledge, this was the first study to investigate the relationship between coffee consumption and the frailty index, which includes both clinical and laboratory assessments. It should be emphasized that the frailty index and frailty phenotype model overlap in the assessment of frailty and that the two are complementary (5). The choice of frailty assessment should depend on the purpose of the assessment, the validity of the instrument, the study population, and the setting in which the assessment is performed (5, 40). The frailty index is characterized by its continuous nature and is more sensitive than the phenotypic model (5, 7). Therefore, the frailty index can more specifically describe the trajectory of health status over time and is more suitable for longitudinal studies. The only prospective study of coffee and frailty used a frailty phenotype model and was conducted in a Spanish population that consumes mostly unfiltered coffee, unlike the United States population (12). Future longitudinal studies are required to determine the relationship between coffee consumption and frailty in different regions, as defined by the frailty index.

However, this study had several limitations. First, this was a cross-sectional study, and the conclusions must be interpreted with caution. Second, coffee consumption and comorbidities were all self-reported, which could result in inaccurate results, although they are the most common methods of collecting data in epidemiological studies. Third, although caffeine played a major role in the observed association, information on filtered or unfiltered coffee was unavailable in the NHANES database. In addition, the number of sweetened coffee and coffee without milk in the database was too small to further examine the effect of sugar and milk on the association between coffee consumption and frailty. Finally, despite controlling for many potential confounders, unknown and unmeasured confounders may still exist.

## 5. Conclusion

We found that caffeinated coffee consumption was independently and nonlinearly associated with frailty risk, especially for non-smokers. However, decaffeinated coffee consumption was not associated with frailty. Future longitudinal studies assessing the association between coffee consumption and frailty are required and should consider using a frailty index. In addition, the underlying mechanisms of this association need to be further elucidated.

## Data availability statement

The original contributions presented in the study are included in the article/Supplementary material, further inquiries can be directed to the corresponding author.

## Ethics statement

The Ethics Review Board of the National Center for Health Statistics approved all NHANES protocols.

## Author contributions

SP, GM, and XZ contributed to the conception and design of the study. SP, YZ, and MD collected and managed the data. SP wrote the first draft of the manuscript. SP, GM, and LB wrote sections of the manuscript. All authors contributed to the article and approved the submitted version.

## Funding

This work was supported by the Science and Technology Development of Henan Province (grant no. 212102310210).

## Conflict of interest

The authors declare that the research was conducted in the absence of any commercial or financial relationships that could be construed as a potential conflict of interest.

## Publisher’s note

All claims expressed in this article are solely those of the authors and do not necessarily represent those of their affiliated organizations, or those of the publisher, the editors and the reviewers. Any product that may be evaluated in this article, or claim that may be made by its manufacturer, is not guaranteed or endorsed by the publisher.

## Supplementary material

The Supplementary material for this article can be found online at: https://www.frontiersin.org/articles/10.3389/fnut.2023.1075817/full#supplementary-material
