# Supplementary material for: Association between coffee intake and frailty among older American adults: A population-based cross-sectional study
Source: Front Nutr. 2023 Feb 2;10:1075817. doi: 10.3389/fnut.2023.1075817 (PMC9932698; doi:10.3389/fnut.2023.1075817)
Supplement: Supplementary file 1 [file Table_1.DOCX]

**Supplement Table 1.** Variables and scorings in the 53-Item Frailty Index

| **Item** | **Scoring** |
| --- | --- |
| **Cognition** | - |
| 1. experience confusion/memory problems | yes=1; no=0 |
| **Dependence** | - |
| 2. managing money difficulty | no difficulty=0; some difficulty=0.33; |
|  | much difficulty=0.66; unable to do=1 |
| 3. walking for a quarter mile difficulty | no difficulty=0; some difficulty=0.33; |
|  | much difficulty=0.66; unable to do=1 |
| 4. walking up 10 steps difficulty | no difficulty=0; some difficulty=0.33; |
|  | much difficulty=0.66; unable to do=1 |
| 5. stooping, crouching, kneeling difficulty | no difficulty=0; some difficulty=0.33; |
|  | much difficulty=0.66; unable to do=1 |
| 6. lifting or carrying difficulty | no difficulty=0; some difficulty=0.33; |
|  | much difficulty=0.66; unable to do=1 |
| 7. house chore difficulty | no difficulty=0; some difficulty=0.33; |
|  | much difficulty=0.66; unable to do=1 |
| 8. preparing meals difficulty | no difficulty=0; some difficulty=0.33; |
|  | much difficulty=0.66; unable to do=1 |
| 9. walking between rooms on same floor | no difficulty=0; some difficulty=0.33; |
|  | much difficulty=0.66; unable to do=1 |
| 10. standing up from armless chair difficulty | no difficulty=0; some difficulty=0.33; |
|  | much difficulty=0.66; unable to do=1 |
| 11. getting in and out of bed difficulty | no difficulty=0; some difficulty=0.33; |
|  | much difficulty=0.66; unable to do=1 |
| 12. using fork, knife, drinking from cup difficulty | no difficulty=0; some difficulty=0.33; |
|  | much difficulty=0.66; unable to do=1 |
| 13. dressing yourself difficulty | no difficulty=0; some difficulty=0.33; |
|  | much difficulty=0.66; unable to do=1 |
| 14. standing for long periods difficulty | no difficulty=0; some difficulty=0.33; |
|  | much difficulty=0.66; unable to do=1 |
| 15. Sitting for long periods difficulty | no difficulty=0; some difficulty=0.33; |
|  | much difficulty=0.66; unable to do=1 |
| 16. reaching up over head difficulty | no difficulty=0; some difficulty=0.33; |
|  | much difficulty=0.66; unable to do=1 |
| 17. grasp/holding small objects difficulty | no difficulty=0; some difficulty=0.33; |
|  | much difficulty=0.66; unable to do=1 |
| 18. going out to movies, events difficulty | no difficulty=0; some difficulty=0.33; |
|  | much difficulty=0.66; unable to do=1 |
| 19. attending social event difficulty | no difficulty=0; some difficulty=0.33; |
|  | much difficulty=0.66; unable to do=1 |
| 20. leisure activity at home difficulty | no difficulty=0; some difficulty=0.33; |
|  | much difficulty=0.66; unable to do=1 |
| 21. push or pull large objects difficulty | no difficulty=0; some difficulty=0.33; |
|  | much difficulty=0.66; unable to do=1 |
| **Depressive Symptoms** | - |
| 22. have little interest in doing things | nearly every day = 1, more than half the days = 0.66 |
|  | several days = 0.33, not at all = 0 |
| 23. feeling down, depressed, or hopeless | nearly every day = 1, more than half the days = 0.66 |
|  | several days = 0.33, not at all = 0 |
| 24. trouble sleeping or sleeping too much | nearly every day = 1, more than half the days = 0.66 |
|  | several days = 0.33, not at all = 0 |
| 25. feeling tired or having little energy | nearly every day = 1, more than half the days = 0.66 |
|  | several days = 0.33, not at all = 0 |
| 26. poor appetite or overeating | nearly every day = 1, more than half the days = 0.66 |
|  | several days = 0.33, not at all = 0 |
| 27. feeling bad about yourself | nearly every day = 1, more than half the days = 0.66 |
|  | several days = 0.33, not at all = 0 |
| 28. trouble concentrating on things | nearly every day = 1, more than half the days = 0.66 |
|  | several days = 0.33, not at all = 0 |
| **Comorbidities** | - |
| 29. doctor ever said you had arthritis | yes = 1; no = 0 |
| 30. ever told you had thyroid problem | yes = 1; no = 0 |
| 31. ever told you had chronic bronchitis | yes = 1; no = 0 |
| 32. ever told you had cancer or malignancy | yes = 1; no = 0 |
| 33. ever told had congestive heart failure | yes = 1; no = 0 |
| 34. ever told you had coronary heart disease | yes = 1; no = 0 |
| 35. ever told you had angina/angina pectoris | yes = 1; no = 0 |
| 36. ever told you had heart attack | yes = 1; no = 0 |
| 37. ever told you had a stroke | yes = 1; no = 0 |
| 38. ever told you had high blood pressure | yes = 1; no = 0 |
| 39. doctor told you have diabetes | yes = 1; no =0; borderline=0.5 |
| 40. ever told you had weak/failing kidneys | yes = 1; no =0 |
| 41. urine leakage bother you? | greatly = 1, very much = 0.75, somewhat = 0.5 |
|  | only a little = 0.25, not at all = 0 |
| **Hospital Utilization and Access to Care** | - |
| 42. general health condition | excellent, very good, good = 0 |
|  | fair, poor = 1 |
| 43. health now compared with 1 year ago | about the same, better = 0 |
|  | worse = 1 |
| 44. overnight hospital patient in last year | yes = 1, no = 0 |
| 45. times receive healthcare over past year | none = 0; 1-4 = 0.5; >=5 =1 |
| 46. number of prescription medicines taken | no = 0; 1-4 = 0.5; >=5 =1 |
| **Physical Performance and Anthropometry** | - |
| 47. body mass index (kg/m^2) | <18.5, ≥30 = 1, 25–<30 = 0.5, 18.5–25 = 0 |
| **Laboratory Values** | - |
| 48. glycohemoglobin(%) | 0%–5.7% = 0, >5.7% = 1 |
| 49. red blood cell count (million cells/ul) | M: 4.7–6.1 = 0, Other = 1 |
|  | F: 4.2–5.4 = 0, Other = 1 |
| 50. hemoglobin (g/dl) | M: 13.5–18 = 0, Other = 1 |
|  | F: 12–16 = 0, Other = 1 |
| 51. red cell distribution width (%) | 11.6–14.6 = 0, Other = 1 |
| 52. lymphocyte percent (%) | 20–40 = 0, Other = 1 |
| 53. segmented neutrophils percent (%) | 40–80 = 0, Other = 1 |

**Supplement Table 2.** Weighted odds ratios (95% confidence intervals) for the association between coffee consumption and frailty stratified by gender

| Caffeinated coffee intake (g/day ) | OR (95%CI )^1^ | P | P for interaction |
| --- | --- | --- | --- |
| **Gender** |  |  | 0.624 |
| **Male** |  |  |  |
| 0 | ref | ref |  |
| 0-253.3 | 0.92(0.69,1.23) | 0.57 |  |
| 253.3-488.4 | 0.75(0.53,1.05) | 0.09 |  |
| >488.4 | 0.73(0.54,0.98) | 0.04 |  |
| **Female** |  |  |  |
| 0 | ref | ref |  |
| 0-253.3 | 0.98(0.76,1.26) | 0.86 |  |
| 253.3-488.4 | 0.89(0.67,1.19) | 0.44 |  |
| >488.4 | 0.62(0.45,0.84) | 0.003 |  |
| **Ethnicity** |  |  | 0.233 |
| **Mexican American** |  |  |  |
| 0 | ref | ref |  |
| 0-253.3 | 1.08(0.68, 1.71) | 0.74 |  |
| 253.3-488.4 | 0.87(0.56, 1.37) | 0.53 |  |
| >488.4 | 0.56(0.25, 1.28) | 0.16 |  |
| **Non-Hispanic White** |  |  |  |
| 0 | ref | ref |  |
| 0-253.3 | 0.97(0.75,1.25) | 0.81 |  |
| 253.3-488.4 | 0.80(0.62,1.04) | 0.09 |  |
| >488.4 | 0.67(0.52,0.87) | 0.003 |  |
| **Non-Hispanic Black** |  |  |  |
| 0 | ref | ref |  |
| 0-253.3 | 0.97(0.71,1.31) | 0.82 |  |
| 253.3-488.4 | 0.67(0.45,0.99) | 0.04 |  |
| >488.4 | 0.69(0.45,1.07) | 0.1 |  |
| **Other Hispanic** |  |  |  |
| 0 | ref | ref |  |
| 0-253.3 | 0.83(0.53, 1.29) | 0.39 |  |
| 253.3-488.4 | 0.75(0.46, 1.23) | 0.25 |  |
| >488.4 | 1.01(0.53, 1.92) | 0.97 |  |
| **Other race** |  |  |  |
| 0 | ref | ref |  |
| 0-253.3 | 1.19(0.48, 2.91) | 0.7 |  |
| 253.3-488.4 | 2.34(0.75, 7.29) | 0.14 |  |
| >488.4 | 0.61(0.19, 1.92) | 0.39 |  |
| **BMI** |  |  | 0.972 |
| **<25** |  |  |  |
| 0 | ref | ref |  |
| 0-253.3 | 1.04(0.69,1.56) | 0.86 |  |
| 253.3-488.4 | 0.77(0.51,1.15) | 0.19 |  |
| >488.4 | 0.61(0.38,0.98) | 0.04 |  |
| **25-30** |  |  |  |
| 0 | ref | ref |  |
| 0-253.3 | 0.94(0.70,1.25) | 0.65 |  |
| 253.3-488.4 | 0.93(0.67,1.29) | 0.65 |  |
| >488.4 | 0.69(0.47,1.02) | 0.06 |  |
| **>30** |  |  |  |
| 0 | ref | ref |  |
| 0-253.3 | 0.99(0.72,1.35) | 0.94 |  |
| 253.3-488.4 | 0.78(0.57,1.06) | 0.11 |  |
| >488.4 | 0.68(0.48,0.95) | 0.03 |  |
| **Physical activity** |  |  | 0.756 |
| **Inactive** |  |  |  |
| 0 | ref | ref |  |
| 0-253.3 | 0.99(0.73,1.32) | 0.92 |  |
| 253.3-488.4 | 0.82(0.58,1.16) | 0.26 |  |
| >488.4 | 0.66(0.47,0.91) | 0.01 |  |
| **Insufficient** |  |  |  |
| 0 | ref | ref |  |
| 0-253.3 | 1.23(0.68,2.24) | 0.49 |  |
| 253.3-488.4 | 1.07(0.60,1.91) | 0.82 |  |
| >488.4 | 0.85(0.45,1.61) | 0.62 |  |
| **Moderate** |  |  |  |
| 0 | ref | ref |  |
| 0-253.3 | 1.15(0.69, 1.92) | 0.59 |  |
| 253.3-488.4 | 1.08(0.59, 1.97) | 0.81 |  |
| >488.4 | 0.64(0.34, 1.21) | 0.17 |  |
| **High** |  |  |  |
| 0 | ref | ref |  |
| 0-253.3 | 0.75(0.54,1.05) | 0.09 |  |
| 253.3-488.4 | 0.70(0.46,1.05) | 0.08 |  |
| >488.4 | 0.62(0.42,0.92) | 0.02 |  |

^1^ adjust for age, gender, BMI, ethnicity, educational level, smoking status, poverty-income ratio, material status, total energy intake, hypertension, diabetes, coronary heart disease, stroke, physical activity.
